# Supplementary material for: SATurn: a modular bioinformatics framework for the design of robust maintainable web-based and standalone applications
Source: Bioinformatics. 2018 Jul 4;35(2):349–51. doi: 10.1093/bioinformatics/bty549 (PMC6329998; doi:10.1093/bioinformatics/bty549)
Supplement: Supplementary Material [file bty549_supplementary_material.docx]

Supplementary Materials

[1 Introduction 1](#_Toc516556093)

[2 Installation instructions 1](#_Toc516556094)

[2.1 Mac OS X 1](#_Toc516556095)

[2.2 Windows 1](#_Toc516556096)

[2.3 Docker 2](#_Toc516556097)

[3 SATurn – User Interface 2](#_Toc516556098)

[4 Example Use: Construct Designer 2](#_Toc516556099)

[4.1 Adding using the table 6](#_Toc516556100)

[4.2 Adding to an existing plate 6](#_Toc516556101)

[4.3 Saving to the SATurn local SQLite database 7](#_Toc516556102)

[5 Extending SATurn with a Python script 8](#_Toc516556103)

# Introduction

The following provides basic instructions for installing SATurn. There follows an example of how to use the construct design tool inside SATurn and a simple example of how to connect a Python script to SATurn. Readers are reminded that the full SATurn manual can be found here: <https://ddamerell53.github.io/SATurn/>.

# Installation instructions

## Mac OS X

- Download <https://github.com/ddamerell53/SATurn/releases/download/v1.0-beta.6/SATurnOSX.app.zip>
- Unzip and click on SATurnOSX.app to launch
- The SATurn installer will then launch
- Read and agree to licenses
- Select your installation directory.
- SATurn will launch automatically but in future you can launch by clicking on Saturn.app which is located in the installation directory you choose above.

## Windows

- Download <https://github.com/ddamerell53/SATurn/releases/download/v1.0-beta.6/SATurn.exe>
- Launch the installer
- Read and agree to licenses
- Select your installation directory.
- SATurn will launch automatically but in future you can launch SATurn by finding it’s entry in your Start menu

Please note that if you receive any firewall prompts regarding NodeJS or Redis you should simply close the prompt windows. This will ensure that SATurn will only be accessible on the PC you installed it upon.

## Docker

- Install Docker if you haven’t already <https://www.docker.com/>
- Open a terminal or command prompt and enter the following commands

docker pull sgcit/saturn:latest
docker run -i -p 0.0.0.0:8091:8091 --name saturn -d sgcit/saturn

- To see the SATurn web-application navigate to: <http://localhost:8091> in a local browser

# SATurn – User Interface

The SATurn user interface, shown below, is designed around the concept that data is loaded into the workspace tree, located on the left of the interface, and is rendered and manipulated using the central panel. At the bottom of the interface is a quick launch tool-bar which can be used to launch various built-in programs like DNA or protein sequence viewers.


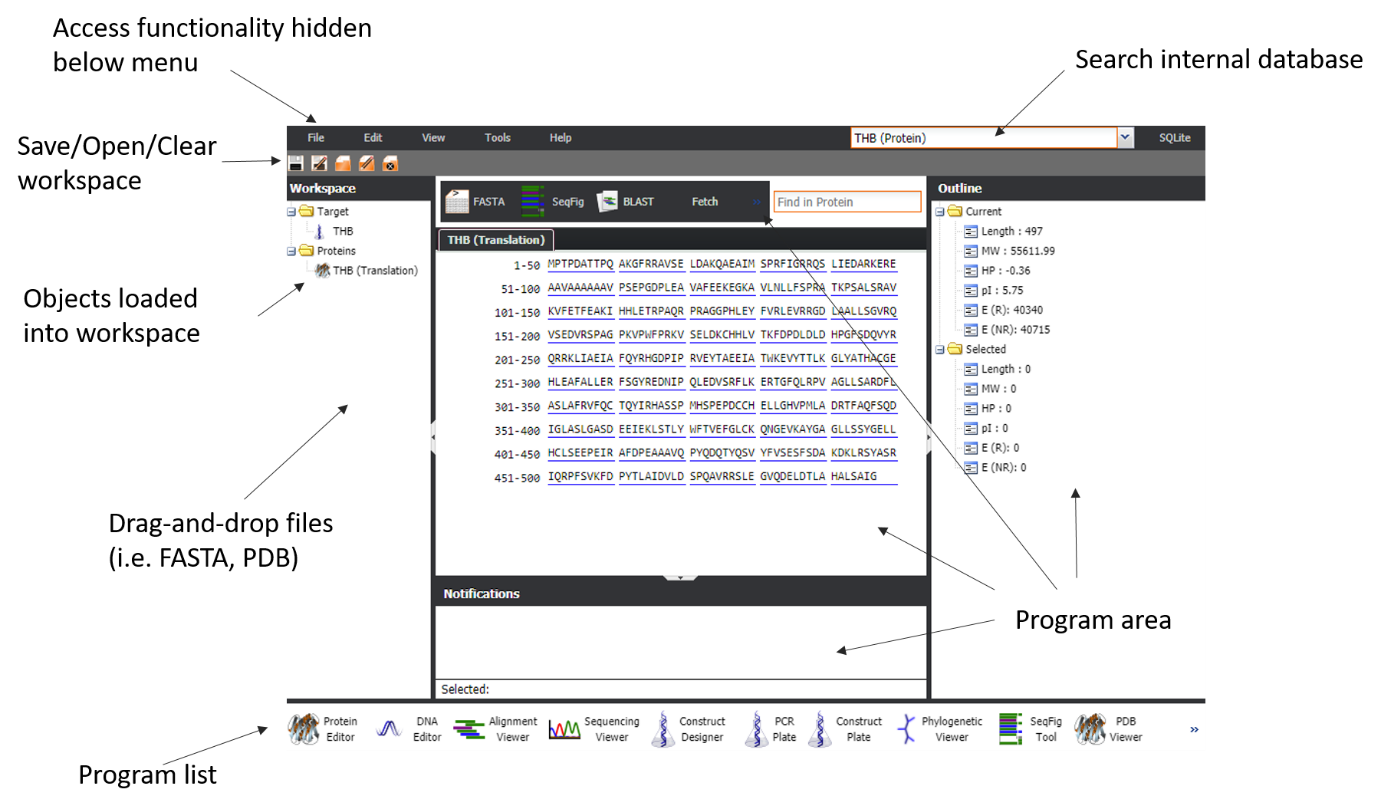


Further details on the user interface can be found in the user manual: <https://ddamerell53.github.io/SATurn/#User%20Guide>

# Example Use: Construct Designer

One of the key uses of the bioinformatics portal created for the SGC using the SATurn framework is to make medium/high throughput cloning conducted at the SGC easier and faster. In this example we describe how to use the construct design tool built into SATurn to create constructs.

The nomenclature used at the SGC for cloning entities is described below

| Workflow | SATurn Entity Name | ID Example | Description |
| --- | --- | --- | --- |
| 1. Select gene | [*Gene*](https://ddamerell53.github.io/SATurn/#Gene) |  | Gene of interest |
| 2. Select isoform | [*Target*](https://ddamerell53.github.io/SATurn/#Target) | BRD1A | Specific isoform |
| 3. Select entry clone | [*Entry clone*](https://ddamerell53.github.io/SATurn/#Entry%20clone) | BRD1A-s001 | Template DNA for PCR |
| 4. Select construct boundaries |  |  |  |
| 5. Enter primers | [*Forward / Reverse Primers*](https://ddamerell53.github.io/SATurn/#Forward%20%2F%20Reverse%20Primers) | BRD1A-f001/r001 |  |
| 6. Enter PCR product | [*Allele*](https://ddamerell53.github.io/SATurn/#Allele) | BRD1A-a001 | PCR product |
| 7. Select vector | [*Vector*](https://ddamerell53.github.io/SATurn/#Vector) |  |  |
| 8. Enter construct | [*Construct*](https://ddamerell53.github.io/SATurn/#Construct) | BRD1A-c001 | Construct |

The following example demonstrates the use of the SATurn construct design tool to create different constructs of the gene TH with varying construct boundaries.

1. Launch SATurn
2. Type THB-s001 into the search box

THB-s001 presents an entry clone / image clone of gene TH

1. Select THB-s001 (Translation)


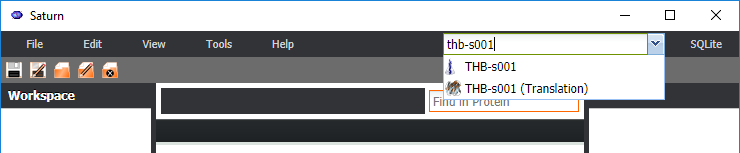


1. The THB-s001 translation will be loaded (as shown below)


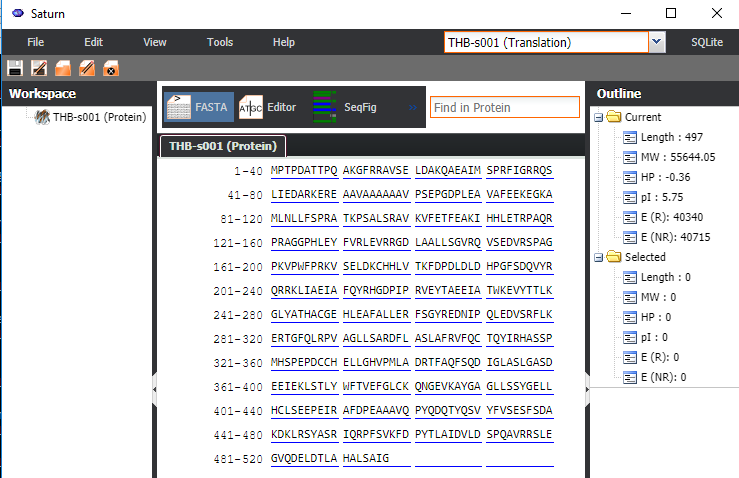


1. Select the region from position 1 to position 200 using the mouse to mark the selection


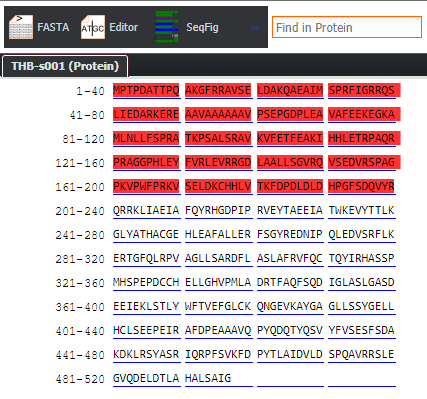


1. Right-click over the selection and select “Add to plate->New plate”


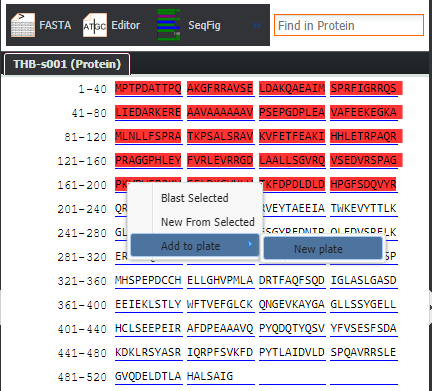


1. This will open a new construct design table.


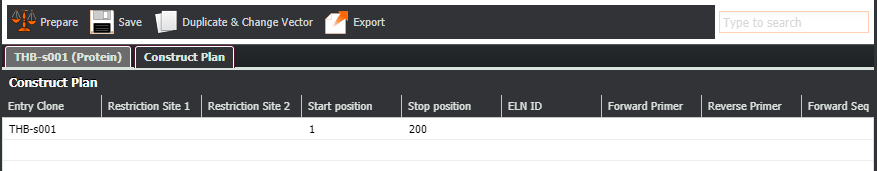


1. Enter pNIC28-Bsa4 into the Vector Name column


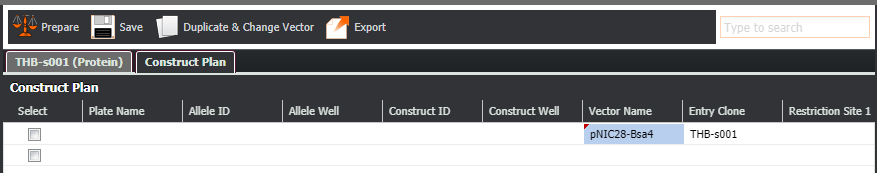


1. Click on “Prepare”


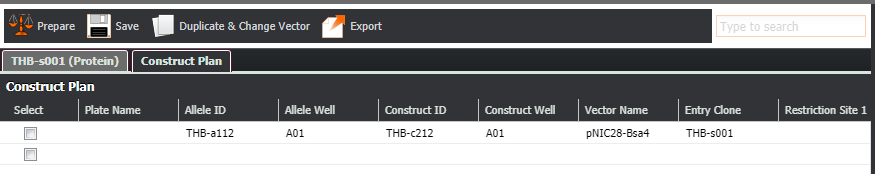


Clicking on “Prepare” automatically generates Allele / PCR product and Construct IDs, assigns positions on the plate and also generates primers and calculates the final DNA and protein sequences for the construct.

Additional constructs can either be created directly using the table or by using the highlight and right-click approach just explained. Both procedures will be explained below.

## Adding a new construct by specifying the start and stop positions directly

1. Scroll along to where you see the start and stop positions recorded for the first construct


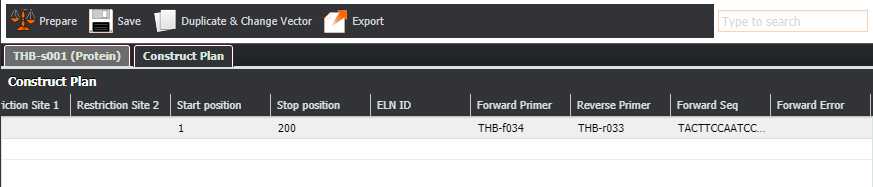


1. In the new row line (row two) enter the following additional start and stop positions: 10 -> 100. Additionally make sure the Vector is set to pNIC28-Bsa4 and the Entry Clone THB-s001


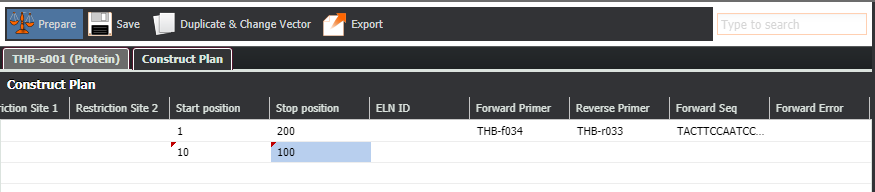


1. Click on “Prepare” to generate IDs and sequences


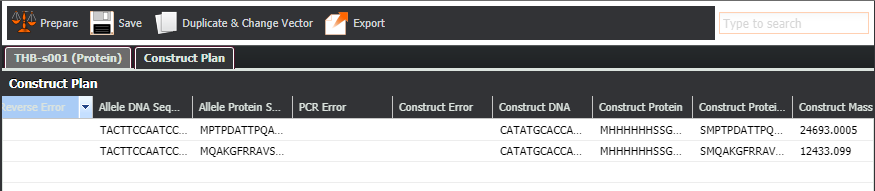


## Adding a new construct to an existing plate

1. Load the entry clone translation you wish to create a construct of
2. Select the right of interest and right-click


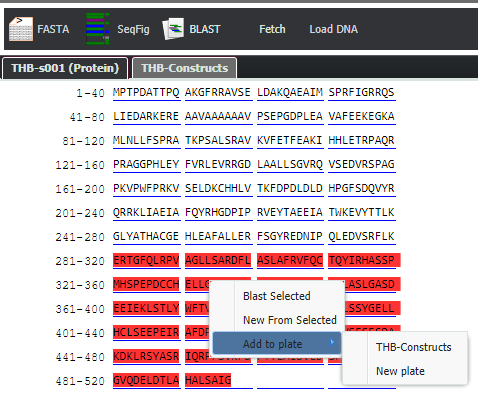


1. Select the name of the plate you wish to add the construct to
2. SATurn will show you the new construct added to the plate.
3. Enter the vector name


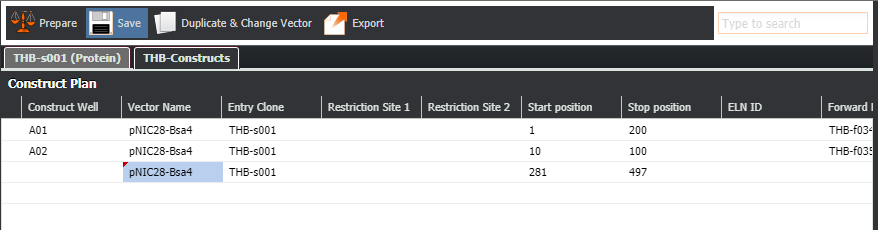


1. Click on “Prepare” to calculate the primers and resultant sequences

## Saving to the SATurn local SQLite database

1. Enter the name of the construct plate into the Plate Name column
2. You can right-click and use the fill-down option to update all rows


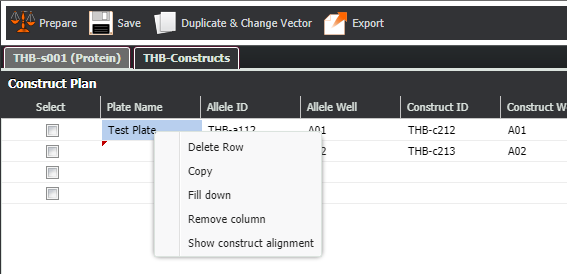


1. Click on the “Save” button

# Extending SATurn with a Python script

Although there are many ways of extending SATurn the simplest way is to add existing scripts via the JSON bridge facility. The following explains how to connect a simple Python script to SATurn – for more detailed information and for examples of other languages see: <https://ddamerell53.github.io/SATurn/#Connecting%20external%20scripts>

1. Within your SATurn installation folder create the following new file

vim build/bin/hooks/example_python_hook.py

import json
import os
import sys

if sys.argv < 2:
 sys.exit('Invalid number of arguments provided')

input_json_path = sys.argv[len(sys.argv)-2]
output_json_path = sys.argv[len(sys.argv)-1]

input_json = None

with open(input_json_path, 'r') as f:
 input_json = json.load(f)

output_json = {'greeting': 'Hello ' + input_json['name']}

with open(output_json_path, 'w') as fw:
 fw.write(json.dumps(output_json))

1. Edit build/services/ServicesLocalLite.json on the server-side and add the following to the “named_query_hooks” section

{
 "name": "saturn.db.provider.hooks.ExternalJsonHook:PythonExample",
 "class": "ssaturn.db.provider.hooks.ExternalJsonHook",
 "method": "run",
 "program": "python",
 "arguments": ["bin/hooks/example_python_hook.py"]
}

1. Launch SATurn and open a scripting editor to test


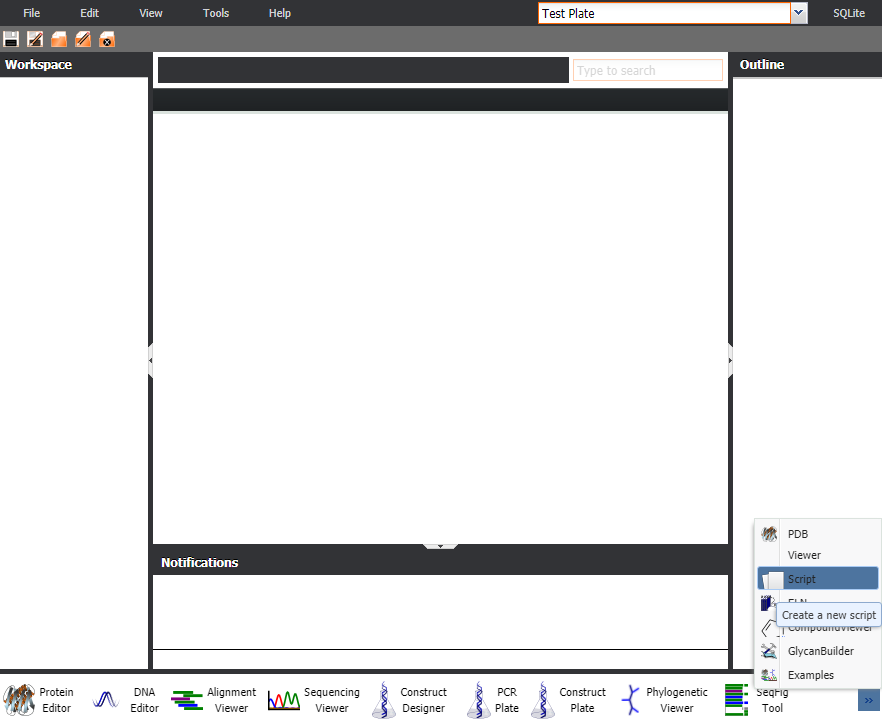


1. Copy and paste the following code into the editor window. Change the “name” parameter value to your name.

saturn.core.Util.getProvider().getByNamedQuery(
 'saturn.db.provider.hooks.ExternalJsonHook:PythonExample',
 [{'name': 'David'}],
 null,
 false,
 function(objs, err){
 if(err != null){
 print(err);
 }else{
 print(objs[0]);
 }
 flush();
 }
);


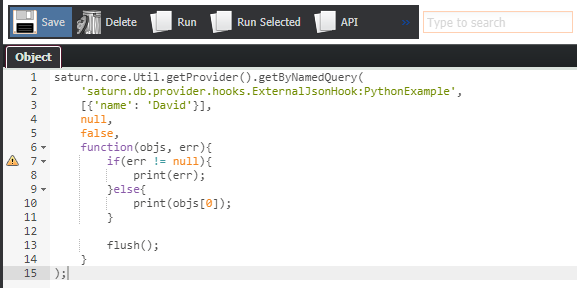


1. Finally click on “Run”
2. Which will print “Hello $name” in the notification box

This is a very basic example but it demonstrates the basics of how to connect a custom external script to SATurn using JSON as a bridge.
